# The effect of multi‐leaf collimator leaf width on VMAT treatment plan quality

**DOI:** 10.1002/acm2.70018

**Published:** 2025-03-13

**Authors:** Gregory Sadharanu Peiris, Brendan Whelan, Nicholas Hardcastle, Suzie Lynn Sheehy

**Affiliations:** ^1^ School of Physics University of Melbourne Melbourne Australia; ^2^ ACRF Image‐X Institute Sydney School of Health Sciences The University of Sydney Sydney Australia; ^3^ Physical Sciences Peter MacCallum Cancer Centre Melbourne Australia

**Keywords:** global access, leaf width, LMIC, multi‐leaf collimator, radiotherapy, VMAT

## Abstract

**Background:**

The advent of volumetric modulated arc therapy (VMAT) in radiotherapy has made it one of the most commonly used techniques in clinical practice. VMAT is the delivery of intensity modulated radiation therapy (IMRT) while the gantry is in motion, and existing literature has shown it has decreased treatment delivery times and the number of monitor units without sacrificing coverage. It has previously been shown that for IMRT, multi‐leaf collimators (MLC) with narrower leaf widths produce demonstrably higher treatment plan quality. However, as VMAT is rapidly becoming the global standard, this needs to be re‐evaluated, especially in a global context. This study assesses the impact of MLC leaf width on VMAT treatment plans and asks whether reducing the number of leaves‐ and thus increasing leaf width‐ provides clinically acceptable treatment plans using VMAT delivery.

**Material & methods:**

Using Varian Eclipse, 51 anonymised patients with prostate, lung, liver, colorectal, or cervical cancer had VMAT treatment plans generated. Treatment plans were generated for MLC leaf widths of 2.5, 5 and 10 mm. Plans were compared using *D*_2_[%], *D*_50_[%], and *D*_98_[%], homogeneity index (HI), conformity index (CI), average leaf pair opening (ALPO), modulation factor (MF) and Estimated Treatment Delivery Time.

**Results:**

The dose to the target structures showed little difference between 2.5 and 5 mm MLC leaves, however 10 mm MLC provided 5% more median dose than the narrower leaf widths for *D*_2_[%] (*p* < 0.05) and *D*_50_[%] (*p* < 0.05). The average HI per leaf width was 0.0777 for 2.5 mm, 0.0752 for 5 mm, and 0.0890 for 10 mm. Organs At Risk (OAR) sparing was consistent between all leaf widths except at low dose percentages, where 10 mm MLC delivered an extra dose to the bladder (*p* < 0.05) and the heart (*p* < 0.05). The average ALPO was 38.0 mm for 2.5 mm, 34.1 mm for 5 mm, and 32.7 mm for 10 mm leaf width. 10 mm MLC leaves traveled a shorter distance from the center (*p* < 0.05). The median MF was 336 MU/Gy for 2.5 mm, 344 MU/Gy for 5 mm, and 384 MU/Gy for 10 mm. There were no differences in estimated treatment delivery time between MLC leaf width.

**Conclusion:**

There is little difference in treatment quality between any of the investigated MLC leaf widths. This work demonstrates that for VMAT treatments, wider MLC leaf widths can still deliver acceptable treatment plans. This finding has potential implications for radiotherapy in low‐ and middle‐income countries and low socio‐economic or rural areas where a focus on MLC robustness and LINAC up‐time is paramount.

## 1 INTRODUCTION

The usage of volumetric modulated arc therapy (VMAT) in radiotherapy has become common place since its inception in 2007.^1^, ^2^ It is now used more frequently than intensity modulated radiation therapy (IMRT) and 3D Conformal (3DCRT) in clinical settings due to its higher efficiency in treatment time and monitor units (MUs).^3^, ^4^, ^5^ VMAT tends to enable more conformal plans compared to IMRT; as such the use of VMAT may change the need for advanced beam shaping technology designed to enhance conformality.

To sculpt therapeutic x‐rays to a target volume, a multi‐leaf collimator (MLC) is used. The MLC consists of independently translated tungsten alloy leaves, perpendicular to the beam's path, that shield parts of the beam. A treatment planning system (TPS) is used to optimize the position of the leaves which move throughout treatment. Over time, the leaf design has tended toward thinner leaves, which allow higher fidelity in collimation and thereby have the potential to provide a higher quality of treatment.^6^ The first MLC that resembled the machines of today were made in 1965 by Toshiba, with 9 pairs of 48 mm wide leaves.^6^, ^7^ Today, leaf widths can be as narrow as 2.5 mm, which results in up to 160 leaves per MLC.^8^, ^9^.

The prevailing understanding is that MLCs with thinner leaves provide a level of target conformity and organs at risk (OAR) sparing that is better than or equal to wider leaves.^10^, ^11^ This is most apparent for small target volumes. Brain tumor treatments achieve better conformity index (CI) and OAR sparing for narrower leaves while requiring fewer MUs.^12^ There is, however, a calculated limit on useful leaf thickness—for a 6 MV beam, the leaf width should not be smaller than 1.5–1.8 mm.^13^ Reducing the leaf width further causes more radiation leakage and a less defined penumbra. For larger tumors, the clinical advantage of thinner leaves is less apparent. While there is no consensus on what constitutes large, target volume has been shown to impact the advantage of thinner leaves. Serna et al. posits volumes larger than 10 cm^3^ constitute large, while Abisheva et al. estimate it to be higher, at around 40 cm^3^.^12^, ^14^ Other studies noted that plans generated for simpler tumor shapes, like those in the liver, spared OAR regardless of MLC leaf width used. Conversely, more complex and concave tumor shapes, like those near the spine, had plans which spared healthy tissue 14%–19% better with thinner leaves.^15^

Within existing literature that examines LINAC downtime and MLC failure modes, failures are more common in MLCs with a greater number of leaves.^16^, ^17^, ^18^, ^19^ For instance, in the 6–12‐month period after an MLC service/clean, Agnew et al. demonstrated (albeit inadvertently) the HDMLC, with narrower leaves, experiencing more hardware and software faults than the standard MLC (wider leaves).^16^ One of the motivations behind Varian's Halcyon design, which includes a simpler MLC with fewer leaves compared to other conventional systems, was to increase reliability and robustness for use in low‐ and middle‐income countries (LMICs) and low‐resource regions.^20^, ^21^, ^22^ While the Halcyon's unique design includes overlapping double‐bank leaves, effectively offering higher resolution, the overall reduction in complexity was intended to reduce the number of potential failure points. As a general engineering principle, the more independent elements present, the lower the reliability will be.^23^ To quote Huang, “it is the active interfaces (i.e., moving surfaces) that are the most common regions for failures to occur.”^24^ Based on this, while narrower leaf widths have been demonstrated to improve treatment quality in some cases, they are also likely to decrease the robustness of the MLC. This necessitates a re‐evaluation of the effect of MLC leaf widths on treatment planning quality using VMAT.

In this work, a treatment planning study was carried out to assess the impact of MLC leaf width on radiotherapy plan quality. Importantly, compared to previous work, this study utilized the modern RT delivery technique of VMAT. In addition, the impact of target volume on treatment plan quality is assessed. We hypothesize that wider leaf widths will show a negligible difference in treatment plan quality as compared to narrower leaf widths as target volume increases. This further research seeks to contribute to the understanding of reliable and robust radiotherapy, with the additional goal of alleviating the challenges faced in cancer care in LMICs, and thus on a global scale.

## 2 MATERIAL AND METHODS

### 2.1 Patient cohort

This project was approved by the Peter MacCallum Cancer Centre Research Ethics Committee. Table 1 outlines the 51 patients who had previously received radiation therapy for cancers of the prostate, lung, liver, rectum, and cervix whose CT and structure data were used in the present work. Anatomical sites were selected based on the World Cancer Report and the Institute for Health Metrics and Evaluation.^25^, ^26^ The patients were selected to give a wide range of target volumes for each anatomical site.

**TABLE 1:** The treatment information for each investigated anatomical site.^23^ ^.^

Anatomical Site	Prostate	Lung	Liver	Colorectal	Cervical
No. of patients	12	10	9	10	10
Fractionation	2 Gy/fx	2 Gy/fx	6–10 Gy/fx	1 Gy/fx	1.8 Gy/fx
No. of fractions	30	30	5	25	25
Avg. tumor size (Range) [cc]	172.8 (110.9−227.2)	492.6 (225.0−670.9)	472.0 (12.1−1459.2)	675.5 (387.5−1024.4)	1045.7 (664.6−2156.8)
No. of arcs	2	2	2	2	2
Beam arrangement	Full rotation	Partial arcs (contralateral lung sparing)	Partial arcs (liver sparing)	Full rotation	Full rotation
Collimator Rot.	15° and 345°	15° and 345°	15° and 345°	15° and 345°	15° and 345°
RapidPlan Model	VPSRG Prostate	VPSRG_RADLUNG	No RapidPlan	VPSRG_RECTUM_15.6	PMCC_Gynae_v1.0

### 2.2 Planning system

VMAT plans were generated using Eclipse (Varian Medical Systems) Photon Optimiser v16.1 for a Varian TrueBeam LINAC. Dose was calculated using AcurosXB reporting dose to medium. The plans were all dual arc with 6 MV photons at a maximum dose rate of 600 MU/min. The MLC leaf widths compared in this study were: 2.5, 5, and 10 mm. The MLCs modeled during treatment planning were:

1. 2.5 mm—120HD^27^
2. 5 mm—Millennium 120^28^
3. 10 mm—Millennium 120

**FIGURE 1:**
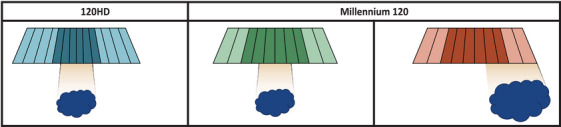
The MLC set up for each leaf width. The 10 mm (right) will use some 5 mm leaves if the target volume is larger than the 10 mm leaf region. MLC, multi‐leaf collimators.

The treatment plan parameters in this study were selected to be identical to the clinically used treatment parameters. Plan objectives and dose constraints were generated using RapidPlan models where available, and where such models were not accessible, plan objectives were duplicated from the clinical plan. Three plans were generated for each patient; using the 120HD MLC (2.5 mm leaves), the central portion of the Millennium 120 (5 mm leaves) and outer portion of the Millennium 120 (10 mm leaves). Plans were normalized according to their clinical protocol: Prostate D_95_ = 98% of prescription dose; Lung, Colorectal, and Cervical D_98_ = 95% of prescription dose; Liver D_99_ = 100% of prescription dose. In the liver patient plans specifically, a small volume within the tumor is typically delivered a dose boost, which is a higher dose than prescribed for the rest of the tumor. This results in an inhomogeneous dose distribution. Regarding the 10 mm leaf plans — because no MLC model which used 10 mm beams was available, the field co‐ordinates were adjusted to use the outer leaves in the Millennium 120 MLC, then the optimizer was executed once with the same objectives. The plans made for the 10 mm leaves used the outer leaves of the Millennium 120 MLC. The 120HD MLC has 2.5 mm wide leaves in the inner 32 leaves and 5 mm leaves for the outer leaves. Jaw tracking, where the position of the jaws within the treatment head are adjusted as MLC aperture changes, is utilized in these treatment plans. This minimizes the dose leakage that can arise from the isocenter being off axis. Targets that exceed 100 mm in any dimension included some 5 mm leaves, as shown in Figure 1. Similarly, for the 120HD MLC targets exceeding 80 mm in any dimension included some 5 mm leaves. The impact of leaf mixing is explored in greater detail in Section 3.3.

### 2.3 Plan evaluation

Plans were evaluated using dose metrics and complexity metrics. Institutional clinical goals based on the eviQ guidelines were used.^29^ The plots for D_98_, D_50_, and D_2_ and dose volume histograms (DVHs) with mean and standard deviation for each anatomical site and leaf width are utilized. Other calculated dose metrics were the homogeneity index (HI), which measures how uniform the dose is across the volume, and CI, which measures target coverage and dose conformity. They are calculated as below,

(1) $$HI=\frac{D_{2}-D_{98}}{D_{50}}\;$$

(2) $$CI=\frac{V_{RI}}{TV}\;$$

where RI is the reference isodose, V_RI_ is the volume of reference isodose, TV is the planned target volume and D_XX_ describes the percentage dose to XX% of the volume.^30^ In general, it is desirable that the HI is as small as possible (a value of 0 indicating a perfectly homogeneous plan), while is it desirable that the CI is as close as possible to 1.

The average leaf pair opening (ALPO) and modulation factor (MF) (MU/Gy) complexity metrics were calculated. Treatment delivery time was estimated by taking the gantry rotation speed at several control points, ω_i_, multiplying the inverse by the sampling interval, ∆θ = 2° and summing from the starting angle, θ_i_, to the final angle, θ_f_, such that time, τ is given by:

(3) $$\tau =\sum_{i=\theta_{i}}^{\theta_{f}}\frac{\Delta \theta }{\omega_{i}}\;$$

A plan is considered as clinically acceptable if it satisfies the clinical goals as adjudged by a clinician.

### 2.4 Statistical methods

Mean DVHs with standard deviations were calculated to visualize the average dose distribution comparing the MLC leaf widths for each anatomical site. Standard deviations were used to depict the variability in dose distribution within each group. Box and Whisker plots were generated to illustrate the distribution of dose metrics, listed above, across the desired MLC leaf widths for each anatomical site and investigate the effect of leaf mixing.

The Wilcoxon signed‐rank test was utilized to determine the statistical significance (or lack thereof) of differences in dose metrics between MLC leaf width. This non‐parametric test was chosen due to the non‐normal distribution of the data. A significance of *α* = 0.05 was used to assess the *p*‐values obtained. Spearman Correlation coefficients were calculated to examine the relationship between target volume and quality metrics (HI, CI) across different anatomical sites and was preferred as it evaluates monotonic relationships between variables. The Kruskal–Wallis test was utilized to investigate leaf‐mixing as it can be calculated with uneven group sizes.

## 3 RESULTS

### 3.1 Dose metrics

#### 3.1.1 Target

**FIGURE 2:**
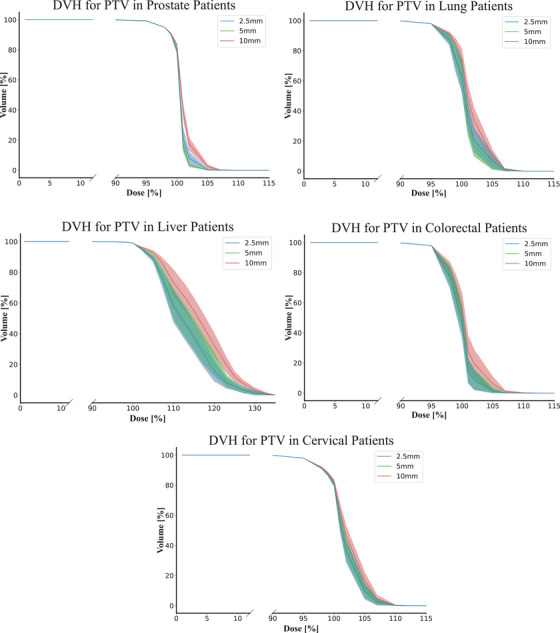
Comparison of the average DVHs for PTV by each leaf width. The different colors denote different leaf widths. The line is the average value and the shaded region is the standard deviation. Note the broken axis for clarity. DVHs, dose volume histograms

**FIGURE A1:**
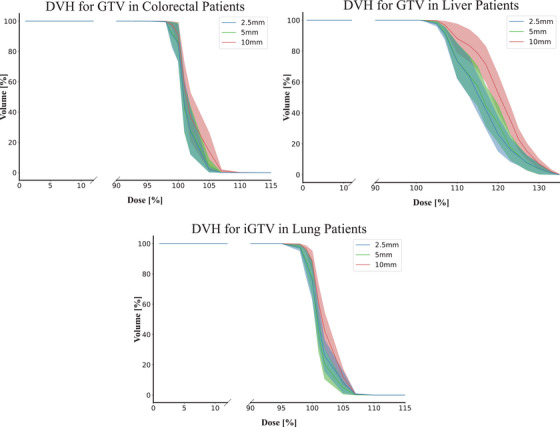
Comparison of the average DVHs for GTV by each leaf width. The different colors denote different leaf widths. The line is the average value and the shaded region is the standard deviation. Note the broken axis for clarity. DVHs, dose volume histograms.

**FIGURE A2:**
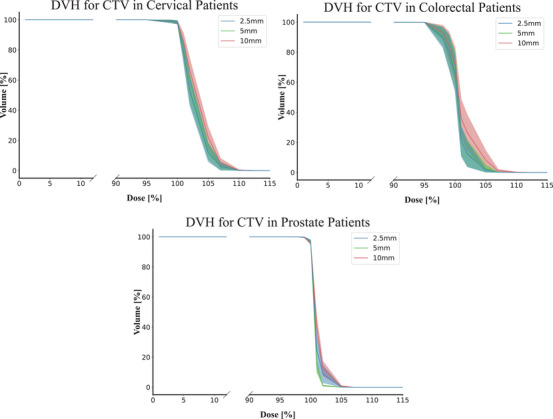
Comparison of the average dose volume histograms for CTV by each leaf width. The different colors denote different leaf widths. The line is the average value and the shaded region is the standard deviation. Note the broken axis for clarity.

The DVHs for the PTV are shown in Figure 2. The DVH for disease sites with a GTV are shown in Figure A1 and disease sites with a CTV are shown in Figure A2. The 10 mm leaves tend to result in higher maximum dose, within one standard deviation, at low volume percentages. The 2.5 and 5 mm MLC leaves deliver a similar average dose with overlapping standard deviation across all treatment sites. All MLCs have a mean dose within one standard deviation of each other for doses delivered to 50% or more of the target volume.

**FIGURE 3:**
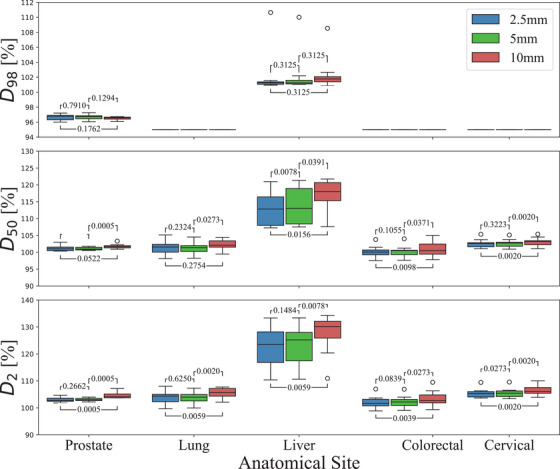
Comparisons of DVH‐based parameters by anatomical site represented by box plots, where the black line indicates the median and the number above each pair indicates the Wilcoxon signed‐rank test *p*‐Value. DVH, dose volume histogram.

The D_98_, D_50_, and D_2_ for each DVH are presented in Figure 3; the Wilcoxon signed‐rank test *p*‐values between each leaf width for selected dose metrics are also shown. All the dose values are fairly stable across leaf widths, with minor increases in dose as the leaf width increases. All the lung, colorectal, and cervical patients have the same D_98_ value, 95%, with no box range since this was the normalization point. The median D_50_ for the 10 mm MLC was 5% higher compared to the 2.5 and 5 mm MLC (*p* < 0.05). The median D_2_ was similarly 5% higher with the 10 mm MLC than the narrow leaf widths (*p* < 0.01). Including the outliers, the range of values is very consistent within each anatomical site, with only the D_2_ values of the 10 mm leaf width delivering higher median dose (*p* < 0.05).

**FIGURE 4:**
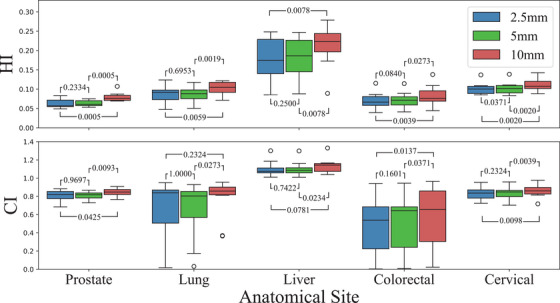
Comparisons of HI (top) and CI (bottom) by anatomical site represented by box plots, where the black line indicates the median. CI, conformity index; HI, homogeneity index.

Examining Figure 2, the liver plans have a prescribed dose over 130%. This is due to planned in‐homogeneities and predetermined dose boosts to volumes within the tumor as mentioned in the methods. This results in the liver plans having higher than average dose compared to other treatment sites, where the average D_98_ to liver patients is 9.24 standard deviations higher than non‐liver patients, 7.8 standard deviations higher for D_50_ and 8.87 higher than D_2_. Dose boosts also affect the target coverage and dose homogeneity, where the HI for liver patients is 4.44 standard deviations higher than non‐liver patients and 1.59 standard deviations higher for CI, as seen in Figure 4.

The distribution of HI and CI for each treatment site is displayed in Figure 4. For non‐liver patients, the achieved homogeneity across different leaf widths demonstrates clinically acceptable medians: specifically 0.0777 for 2.5 mm, 0.0752 for 5 mm, and 0.0890 for 10 mm. The ranges observed for leaf width exhibit remarkable similarity to one another. The CI median and range across all anatomical sites is consistent. The optimal value for CI is 1 as per Equation 2. Liver plans that have a CI over 1 are due to dose boosts delivering over 100% dose to the target volume.

#### 3.1.2 OAR

**FIGURE 5:**
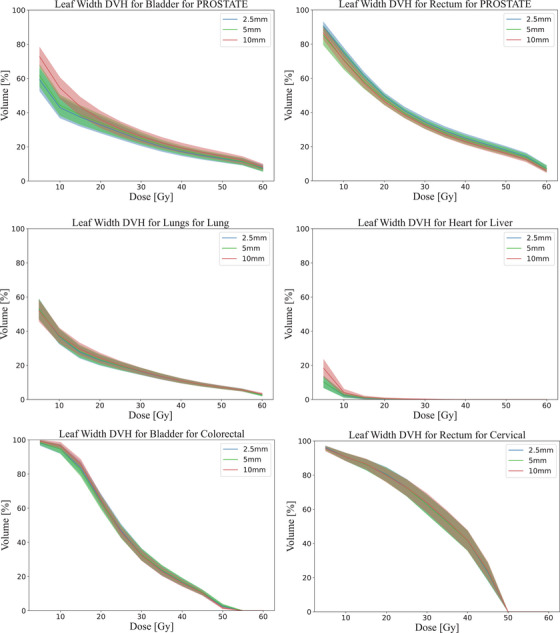
Comparison of the average DVHs for selected OAR by each leaf width per anatomical site. DVHs, dose volume histograms; OAR, organs at risk.

**FIGURE B1:**
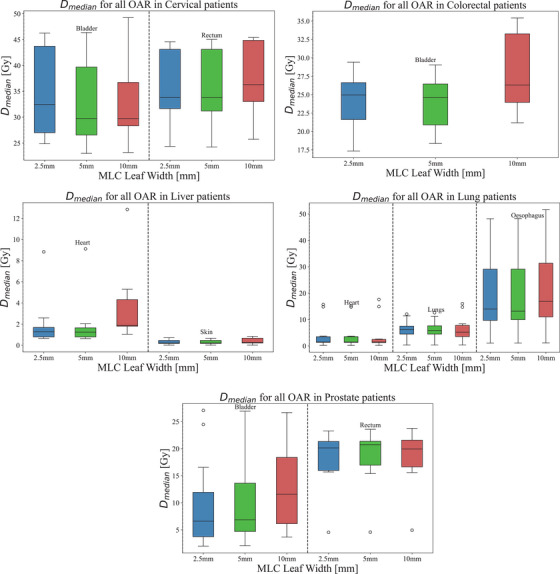
Comparing the median dose to OAR as delivered by different MLC leaf widths per anatomical sites.

**FIGURE B2:**
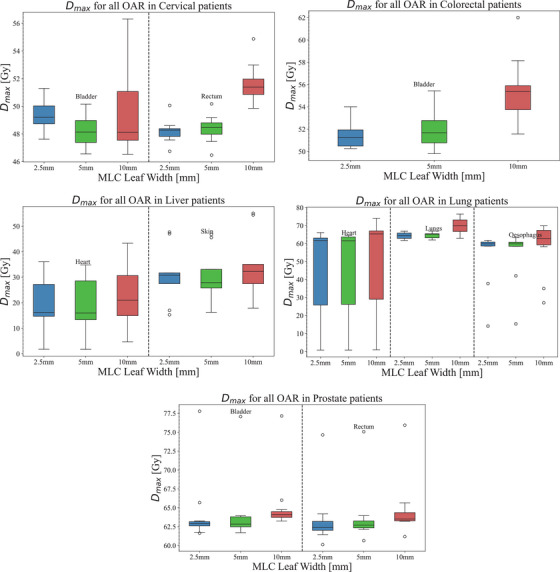
Comparing the max dose to OAR as delivered by different MLC leaf widths per anatomical sites.

A selection of the OAR's DVHs is displayed in Figure 5. Figure B1 contains boxplots of D_median_ and Figure B2 displays boxplots of D_max_ for all relevant OARs per leaf width. Notably, a higher volume receives low to mid range doses with the 10 mm MLC within the bladder (*p* < 0.001) and the heart (*p* < 0.05), though it is still within a clinically acceptable range. In cervical and lung patients, the OAR sparing is near identical across all leaf widths. Between the 2.5 and 5 mm, the 5 mm delivers marginally less dose in the rectum OAR for prostate patients at V5Gy (*p* = 0.9102) and V60Gy (*p* = 0.5703).

### 3.2 Plan complexity metrics

**FIGURE 6:**
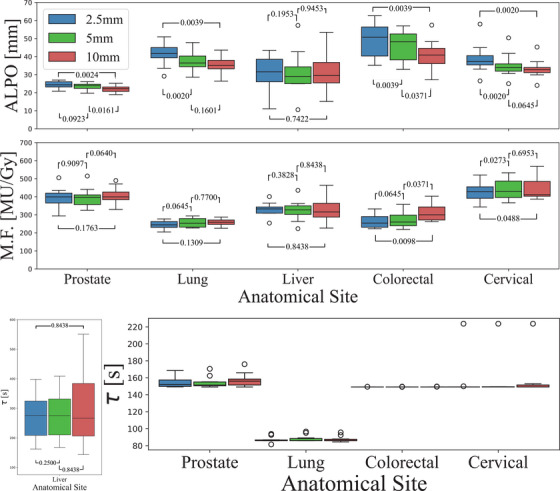
Box and whisker plots comparing the plan complexity metrics of ALPO (top), MF (MU/Gy) (centre) and estimated treatment time (bottom) for different leaf widths. The black bar is the median value. ALPO, average leaf pair opening; MF, modulation factor.

The plots in Figure 6 compare plan complexity metrics and estimated treatment time for different leaf widths across each investigated treatment site. The ALPO is indicative of the distance traveled by the leaves during delivery. This metric can also serve a surrogate for time‐to‐failure, as based on Huang's statement.^24^ The median values for ALPO decrease as leaf width increases across all sites except liver, despite treating the same target volume. The averages are 38.0 mm for 2.5 mm, 34.1 mm for 5 mm and 32.7 mm for 10 mm leaf width. Thus the 10 mm MLC leaves retract into the leaf bank by a shorter distance overall (*p* < 0.0002). However, noting that MLCs with narrower leaf widths will have more leaves, the same ALPO in narrower leaves implies greater usage and attrition.

Similar to ALPO, the difference in MF between the leaf widths is minimal. The median MF is 336 MU/Gy for 2.5 mm, 344 MU/Gy for 5 mm and 384 MU/Gy for 10 mm. This is further detailed in Section 4.

The final metric investigated was a treatment delivery time estimate, *τ*, in the bottom panel of Figure 6. The median times demonstrate consistent values across treatment site, with no significant variations observed. The proximity of these values, within a 10s range, indicates comparable durations for radiation delivery across the evaluated cases. Once again, liver targets behave demonstrably differently, with a larger range and higher median values. (Figure C3)

#### 3.2.1 Plan quality variation

**FIGURE 7:**
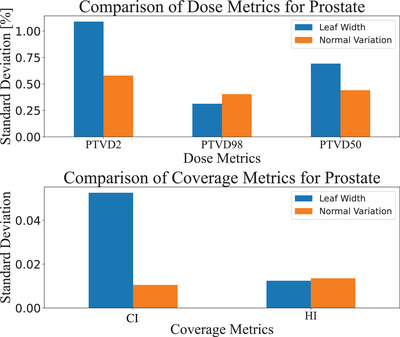
A comparative plot between the standard deviation due to normal variation in treatment planning to the variation encountered in the leaf width comparative study for prostate cases.

**FIGURE C1:**
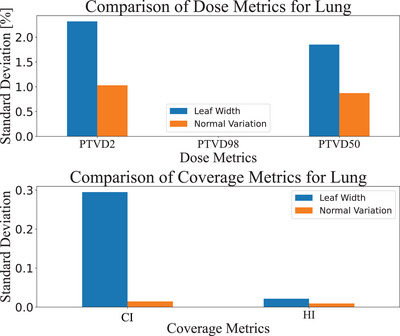
A comparative plot between the standard deviation due to normal variation in treatment planning to the variation encountered in the leaf width comparative study for Lung cases.

**FIGURE C2:**
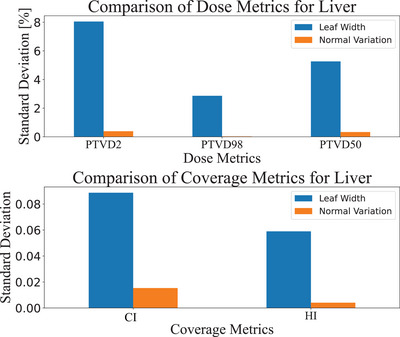
A comparative plot between the standard deviation due to normal variation in treatment planning to the variation encountered in the leaf width comparative study for Liver cases.

**FIGURE C3:**
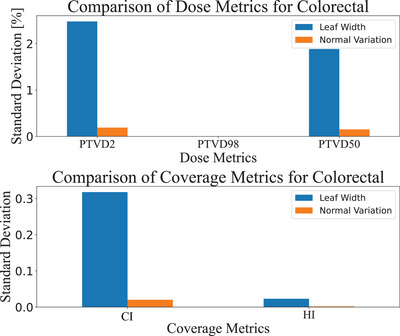
A comparative plot between the standard deviation due to normal variation in treatment planning to the variation encountered in the leaf width comparative study for Colorectal cases.

**FIGURE C4:**
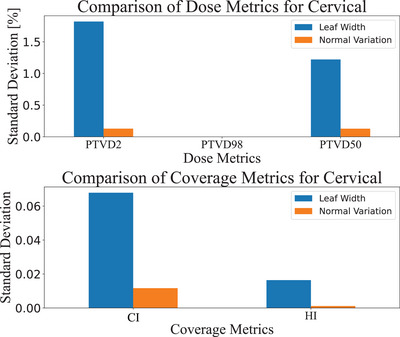
A comparative plot between the standard deviation due to normal variation in treatment planning to the variation encountered in the leaf width comparative study for Cervical cases.

One method that was considered to assess the acceptability of a treatment plan for differing MLC leaf widths was to compare the variation in leaf width to the normal variation in plan quality for a given disease site. If the variation from a valid simulation for the same target and leaf model was larger than the variation between leaf widths, this would class it as inherently clinically acceptable. This option was explored by repeating the simulation for a given target with the 5 mm MLC 10 times, to keep a consistent leaf model, and comparing the standard deviation against the standard deviation of all results from the leaf width comparison. The standard deviation of natural variation is consistently smaller than the standard deviation of all sites and metrics, except for D_98_ and HI for Prostate, as displayed in Figure 7. Comparative plots for Lung (Figure C1), Liver (Figure C2), Colorectal (Figure C3) and Cervical (Figure C4) plans are made available in Appendix C.

### 3.3 Volume dependence

#### 3.3.1 Impact of volume of coverage

**FIGURE 8:**
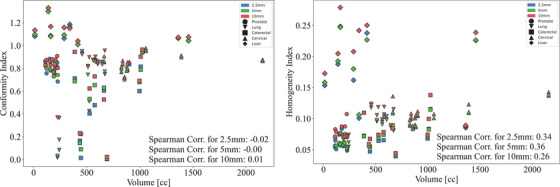
Scatter plots detailing the relationship between volume and CI (left) and HI (right) for each investigated leaf width with the Spearman co‐efficient listed. CI, conformity index; HI, homogeneity index.

The dependence of coverage metrics on volume of the PTV are displayed in Figure 8. Note that typical volumes of tumors depend on the geographic and socio‐economic region in which they are diagnosed: diagnosis of cancers in LMICs typically happen at advanced stages, resulting in larger tumors on average, as introduced in Section 1. Figure 8 displays the relationship with target conformity, dose homogeneity and volume. CI is mostly grouped above and around the 0.8 mark. Values less than this correspond to smaller lung and colorectal volumes. The wider leaf widths have CI closer to 1 and consistently have a higher CI than narrower leaves. There was no correlation between CI and tumor volume for the 2.5 mm MLC (*r* = −0.02, *p* = 0.8976), the 5 mm MLC (*r* = 0.00, *p* = 0.9966) or the 10 mm MLC (*r* = 0.01, *p* = 0.9427). (Figure C4)

HI increases with volume for 2.5 mm MLC (*r* = 0.34, *p* = 0.0153), 5 mm MLC (*r* = 0.36, *p* = 0.0100) and 10 mm MLC (*r* = 0.26, *p* = 0.0718). In Figure 8, the wider leaves result in a higher HI than narrower leaves, congruent with Figure 4. The correlation is lower for 10 mm MLCs suggesting a lower dependence on volume with wider leaves than with narrower MLC leaves. The prostate cases consistently demonstrate superior treatment metrics across all leaf widths in terms of both CI and HI. Other sites vary depending on the leaf width utilized. This suggests that neither wider leaves nor narrower leaves provide a higher quality of treatment overall, but rather an appropriate contextual consideration of MLC leaf width can improve treatment plan quality.

#### 3.3.2 Effect of MLC leaf mixing

**FIGURE 9:**
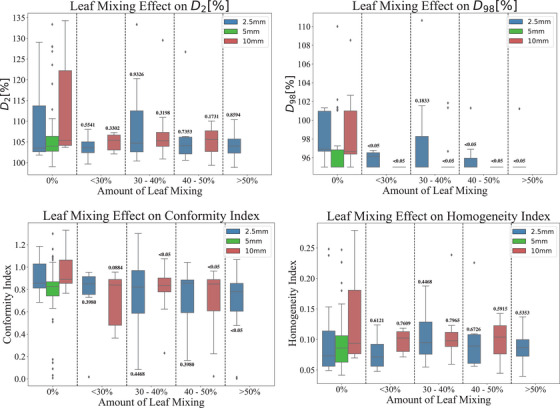
Box and Whisker Plots comparing the effect of varying amounts of leaf mixing on dose metrics, D2 (top left) and D98 (top right) and coverage metrics, Conformity (bottom left) and Homogeneity (bottom right). The number above indicates the Kruskal–Wallis test between the amount of leaf mixing and 0% leaf mixing.

One of the main limitations in this study is the distinct lack of MLCs which have a constant leaf width. A characterization of the effect of MLC leaf width mixing is presented in Figure 9. In this study, to quantify the degree of leaf mixing within Varian Eclipse treatment plans, where no built‐in function is available to directly assess the use of MLC leaves, an approximation method was developed. Due to the complexity introduced by jaw tracking, which dynamically adjusts the field size throughout the treatment delivery, a static approach was adopted. The maximum field size observed during treatment was identified, and the distribution of MLC leaf widths was analyzed within this field. Specifically, the number of leaves of each width (e.g., 2.5 and 10 mm) was recorded, and the proportion of MLC leaves was calculated relative to the total number of leaves used. This method approximates the extent of leaf mixing by representing the contribution of different leaf widths as a percentage, with a higher proportion of 5 mm leaves indicating greater leaf mixing. While not an exact measure, this approximation provides a reasonable estimate of MLC leaf mixing and its potential impact on treatment plan complexity.

In the instance of D_2_ and Homogeneity, the median values are not statistically distinct from 0% leaf mixing. The results from the Kruskal–Wallis test reveal that for the D_98_ metric, there are statistically significant differences between the 0% leaf mixing bin and the other leaf mixing categories for both 2.5 and 10 mm MLC plans. Conversely, the CI shows no statistically significant differences for leaf mixing values below 50% in the 2.5 mm MLC plans and below 30% in the 10 mm MLC plans. This suggests that, up to these thresholds, leaf mixing has minimal impact on CI in these respective plans.

Additionally, the D_98_ results for 2.5 mm MLC plans show no statistically significant difference from the 0% leaf mixing bin in the 5 mm MLC plans, indicating that as more 5 mm leaves are incorporated, the D_98_ results begin to converge with those produced by the 5 mm MLC configuration. Similarly, the CI for higher levels of leaf mixing becomes statistically indistinguishable from the CI of the 5 mm MLC plans, implying that as leaf mixing increases, the influence of the 5 mm MLC becomes more prominent in shaping the results, particularly in terms of CI. These findings suggest that the inclusion of a greater number of 5 mm leaves has a significant effect on D_98_ and CI, with the results increasingly reflecting those typically observed with the 5 mm MLC system.

## 4 DISCUSSION

In this study, we compared the effect of the MLC leaf widths 2.5 mm, 5, and 10 mm on VMAT treatment plan quality. For most metrics, there was little difference between the 2.5 and 5 mm leaf width. D_50_ and D_2_ values for 2.5 and 5 mm are within 2% of each other for all anatomical sites. The only instance with a marginal difference between the two is in the rectum DVH where the 2.5 mm delivers a slightly higher dose, though this is entirely within one standard deviation. The 10 mm leaf widths generally deliver slightly more unwanted dose than the 2.5 and 5 mm, however, it is still in a clinically acceptable range.

To ensure that natural variations in treatment plan quality were minimized to highlight the effect of leaf width, several methods were put in place. First, multiple patient plans from each anatomical site were examined to minimize random variation. This study also uses the same dose calculation algorithm for all disease sites as the current literature observes significant variation in D_max_, D_min,_ D_95,_ and HI where different dose calculations algorithms were used.^31^ Furthermore, the utilization of RapidPlan knowledge‐based treatment planning that uses Machine Learning guarantees a level of consistency in plan quality where stochastic delivery errors have been found to be clinically acceptable.^32^

In the present study, we chose to optimize all plans with the same objective function weightings in order to maintain consistency across all parameters except for MLC leaf width. This is different to clinical practice, in which objectives and constraints would be adjusted to satisfy all clinical protocols before the delivery of a treatment plan. As such, it is possible that the minor differences in quality and complexity observed could be recovered in a clinical setting. This would be an interesting avenue for future study. The 10 mm plans in Figure 2 demonstrate consistently hotter plans, however, these can be normalized to ensure clinical objectives are met. Here the differences are visible since we are aiming for a direct comparison with minimal variables, whereas in practice, the plans would be normalized or the optimization refined to achieve the desired plan.

Non‐uniform dose delivery has been demonstrated in recent literature to have no effect and in some cases improve treatment.^33^, ^34^, ^35^, ^36^ For some anatomical sites, the uniformity of dose case been called into question.^37^ Specifically, Craft et al. mentions prostate and liver as “disease sites where there is no motivation to spare anything inside the target.” This impact of hotter or homogenous plans on colorectal and cervical tumors could not be ascertained from existing literature. For a clinical plan, it may be preferable to normalize or further optimize to avoid the risk of a hot spot shifting from PTV to OAR due to anatomical shift or set up errors. This additional step was not included in the present study as it may affect the outcomes of the variables under study but would be included in a clinical treatment plan.

The approach described above allows for a direct MLC leaf width comparison between the different MLCs. One of the limitations this study encounters is the lack of a readily available 10 mm MLC within the TPS. Two sites (prostate, liver) with smaller tumor volumes relied solely on the 10 mm and 2.5 mm leaves, and results for these sites are expected to be valid. However, the limited span of the 10 mm MLC region within the Millennium 120 MLC, and 2.5 mm region within the 120HD MLC meant that the sites with larger typical volumes (lung, colorectal, and cervical) had treatment plans, which incorporated some 5 mm leaves as well, since the volumes were too large for a single leaf width region alone. Information regarding the exact percentage of tumor volume treated by each leaf width is not available from the TPS so we estimate this using the maximum field size from the Beam Eye View cross section. The complexity of characterizing the effect of leaf mixing arises from the volume dependence discussed in Section 3.3 as the larger a volume is, the greater the amount of leaf mixing. Since this study is not controlling for volume or shape complexity, the results in Figure 9 are preliminary, though serve to characterize the impact of multiple leaf types on the other results we have presented. We are however, satisfied that results across all sites remain valid, as the larger volumes where this limitation occurs require less fidelity, as evidenced in existing literature for IMRT.^12^, ^14^ Future studies should explore the use of a TPS with VMAT optimization capabilities for a 10 mm MLC, which is not available in Eclipse.

One growing context in which wider MLC leaves can prove to be more beneficial is in LMICs. Despite sparse data, the MLC is known to cause frequent faults and prolonged LINAC downtime in LMICs.^38^, ^39^ Over 50% of the mechanical faults in a LINAC and over 50% of component replacements for LINAC repair are due to issues within the MLC.^38^ The MLC downtime is seven times longer in LMICs than in high income countires (HICs) due to a confluence of factors, where limited access to spare parts and increased patient load of trained technicians in LMICs are the primary reasons. Using principles of reliability engineering and some preliminary evidence in MLC maintenance studies, fewer MLC leaves, and therefore, wider leaves, can increase MLC reliability. This study shows that, with VMAT, fewer leaves do not come at the expense of treatment plan quality. Prior findings have shown that while 5 mm MLC leaves result in superior dose conformity and reduced dose toxicity, it is in the context of brain lesions and head and neck cancers. Previous studies investigating cancers commonly found in LMICs showed no clinical difference between narrower and wider leaves for non‐VMAT plans.^11^, ^15^, ^40^, ^41^ This study affirms past findings while contributing to the recent clinical technique, VMAT.

One arising issue is how the LMIC caseload might evolve over time: even if a 5 mm or 10 mm leaf width increases robustness and delivers high levels of dose conformity, in the future, with better early detection and smaller metastases, a 2.5 mm leaf width would be preferred. In these situations, it is possible to mimic a 2.5 mm leaf width using a 5 mm MLC. This is done by shifting the isocenter by half a leaf width (2.5 mm) between arcs, as done by Park et al. to imitate an extremely narrow (1.25 mm) leaf width.^42^ Park showed the shifted plans had better HI and CI to the target than the original 2.5 mm. A similar approach could be utilized to emulate 5 mm leaf widths with a 10 mm MLC. This would be another interesting avenue for future study.

Although this discussion is made in the context of LMICs, it has the potential to be beneficial to low‐resource settings more broadly. While HICs generally have better access to spare parts and trained technicians, regions within HICs which do not for example, rural areas, low socio‐economic areas etc. might stand to benefit from a more robust LINAC. However, there is no existing literature on RT LINAC and MLC breakdowns in these regions. This motivates studies in HICs to assess whether this method could benefit HICs as well as LMICs.

## 5 CONCLUSION

We investigated the treatment quality for plans using different MLC leaf widths by comparing treatment plan metrics for an LMIC oriented caseload. The results for the 5 mm and 2.5 mm leaf widths were comparable, with no significant advantage from narrower leaves demonstrated. The 10 mm does deliver slightly more unwanted dose, but in most cases, this was not considered clinically significant.

For the cohort investigated, the key finding of this study is that clinically acceptable VMAT treatment plans could be generated using wider MLC leaves to a similar degree to the narrower MLC leaves in current use. This finding may have important implications for the design of more robust radiotherapy machines for use in LMIC environments.

## AUTHOR CONTRIBUTIONS

Gregory Sadharanu Peiris: study concepts and design, literature research, experimental studies / data analysis, statistical analysis, manuscript preparation, manuscript editing Brendan Whelan: study concepts and design, manuscript preparation, manuscript editing Nicholas Hardcastle: guarantor of integrity of the entire study, study concepts and design, manuscript preparation, manuscript editing Suzie Lynn Sheehy: guarantor of integrity of the entire study, study concepts and design, manuscript preparation, manuscript editing.

## CONFLICT OF INTEREST STATEMENT

Dr. Brendan Whelan consults for tibaray, a startup developing next generation radiotherapy.

## Data Availability

The data that support the findings of this study are available from the corresponding author upon reasonable request.

## APPENDIX A

### DVHs

I.I GTV

I.II CTV

## APPENDIX B

### DOSE BOX PLOTS FOR OARS

II.I D_median_ FOR OAR

II.II D_max_ FOR OAR
